# The Preclinical and Clinical Progress of Bacteriophages and Their Lytic Enzymes: The Parts are Easier than the Whole

**DOI:** 10.3390/v11020096

**Published:** 2019-01-24

**Authors:** Karim Abdelkader, Hans Gerstmans, Amal Saafan, Tarek Dishisha, Yves Briers

**Affiliations:** 1 Laboratory of Applied Biotechnology, Department of Biotechnology, Ghent University, Valentin Vaerwijckweg 1, B-9000 Ghent, Belgium; karimabdelkadersoufi.abdelkader@ugent.be (K.A.); hans.gerstmans@ugent.be (H.G.); 2 Department of Microbiology and Immunology, Faculty of Pharmacy, Beni-Suef University, Beni-Suef 62511, Egypt; amaleisa_sb@yahoo.com (A.S.); tarek.dishisha@pharm.bsu.edu.eg (T.D.); 3 MeBioS-Biosensors group, Department of Biosystems, KU Leuven, Willem de Croylaan 42, B-3001 Leuven, Belgium; 4 Laboratory of Gene Technology, Department of Biosystems, KU Leuven, Kasteelpark Arenberg 21, B-3001 Leuven, Belgium; 5 Department of Pharmaceutical Microbiology, Faculty of Pharmacy, Menoufia University, Shebin ElKoum 51132, Egypt

**Keywords:** phage, lytic enzyme, endolysin, antibacterial, antibiotic, preclinical analysis, clinical trial

## Abstract

The therapeutic potential of phages has been considered since their first identification more than a century ago. The evident concept of using a natural predator to treat bacterial infections has, however, since then been challenged considerably. Initially, the vast success of antibiotics almost eliminated the study of phages for therapy. Upon the renaissance of phage therapy research, the most provocative and unique properties of phages such as high specificity, self-replication and co-evolution prohibited a rapid preclinical and clinical development. On the one hand, the typical trajectory followed by small molecule antibiotics could not be simply translated into the preclinical analysis of phages, exemplified by the need for complex broad spectrum or personalized phage cocktails of high purity and the more complex pharmacokinetics. On the other hand, there was no fitting regulatory framework to deal with flexible and sustainable phage therapy approaches, including the setup and approval of adequate clinical trials. While significant advances are incrementally made to eliminate these hurdles, phage-inspired antibacterials have progressed in the slipstream of phage therapy, benefiting from the lack of hurdles that are typically associated with phage therapy. Most advanced are phage lytic enzymes that kill bacteria through peptidoglycan degradation and osmotic lysis. Both phages and their lytic enzymes are now widely considered as safe and have now progressed to clinical phase II to show clinical efficacy as pharmaceutical. Yet, more initiatives are needed to fill the clinical pipeline to beat the typical attrition rates of clinical evaluation and to come to a true evaluation of phages and phage lytic enzymes in the clinic.

## 1. Phages and Phage-Inspired Antibiotics

Ever since their discovery, bacteriophages have inspired to be used as antibacterial therapeutics. Whereas initially the use of intact phages has been considered for therapy, intensive research of phage biology has nowadays yielded several other avenues of investigation towards the development of novel antibacterials. Indeed, during their replication cycle phages interfere at all stages with the bacterial integrity and viability, providing different clues for novel antibacterials.

Many phages are equipped with polysaccharide depolymerases in their tail fibers or tail spikes. When initiating a phage infection cycle, these enzymes degrade capsule polysaccharides (CPSs), O-polysaccharide chains of lipopolysaccharide (LPS) molecules or extracellular polysaccharides (EPSs) that form a biofilm matrix [1]. Treatment of mice infected with *Klebsiella pneumoniae* with a capsule-specific depolymerase led to complete survival without significant clinical signs of illness, whereas the lack of treatment resulted in a high lethality (87.5%) [2]. Moreover, isolated phages equipped with putative depolymerases or their isolated depolymerases successfully rescued mice and *Galleria mellonella* larvae infected by a hypervirulent *K. pneumoniae* strain, which is a hyper-producer of capsular polysaccharide [3,4,5,6,7]. Additionally, *Escherichia coli* K1, K5 and K30-specific depolymerases were successfully evaluated in a mouse thigh model to treat infections [8]. Depolymerases are proposed to function as antivirulence compounds through the degradation of a major bacterial virulence factor. Encapsulated *K. pneumoniae* cells exposed to recombinant capsular depolymerases become more prone for complement-mediated killing in serum and phagocytosis, resulting in a reduced virulence in a *Galleria mellonella* larvae infection model [4]. In addition, capsule removal by depolymerases can increase the in vivo efficacy of standard-of-care antibiotics [9].

An important class of phage-encoded enzymes with antibacterial potential are phage lytic enzymes [10,11]. To eject the phage genome into the host cell, phages locally degrade the cell wall with a first phage lytic enzyme, called virion-associated peptidoglycan hydrolase. This enzyme creates a local hole in the peptidoglycan layer for transfer of the genome, but its association with the phage particle structure avoids extensive damage of the peptidoglycan layer taking place. A second phage lytic enzyme is produced at the end of the replication cycle. This protein is produced as a soluble, free enzyme and is called an endolysin. At a genetically programmed time point, (pin)holins release the endolysin to the periplasm or activate previously secreted endolysin molecules. These endolysins then extensively degrade the peptidoglycan layer from within, resulting in a sudden osmotic lysis of the bacterial cell and dispersion of the newly matured phage particles [12]. The potential of phage lytic enzymes as antibacterials for use in medicine and food conservation was first described in 2001 and 2000, respectively [13,14].

Besides intact phages and phage-encoded enzymes, also small chemical molecules mimicking growth-inhibitory phage-host interactions have been proposed as novel antibacterials. From the early stage of infection, such phage-host interactions take place to control the host cell machinery and to redirect the cellular resources for phage production. These interactions are often mediated by small proteins, which are among the earliest expressed ones [15]. Thirty-one phage proteins were identified in 26 *Staphylococcus aureus* phages with a growth-inhibitory effect [16]. The specific interaction between a phage protein and the bacterial target DnaI was used to screen for small molecules, mimicking the effect of the phage protein. Insights in the basic biology of phage-host interactions are now drastically accumulating [17,18,19,20], offering further clues for small molecule design.

The first phage-borne depolymerases are now evaluated preclinically, while phage-inspired antibiotics based on phage-host interactions are in the discovery phase with a single small molecule hit selected. In contrast, both phages and lytic enzymes have been demonstrated to be efficient and safe in extensive preclinical studies [21,22,23,24,25]. In addition, the safety of specific phages and lytic enzymes has been proven in human case studies and completed clinical trials phase I (Table 1 and Table 2). One phase I/II and one phase I trial has been completed with static phage-containing medicinal products (drugs in US), and three phase I trials for phage lytic enzymes. Different clinical phase II or II/III trials have been initiated for phages and phage lytic enzymes. Phage-containing medicinal products (drugs) and recombinant phage lytic enzymes are thus the most advanced phage(-derived) products on the clinical development path for use in human medicine. Both phages and phage lytic enzymes were withheld in a recent pipeline review of alternative antibacterials [26]. Phage lytic enzymes were classified as the alternative with the highest potential on effective implementation for antibacterial therapy. Wild type and engineered phages were also scored high for their potential impact, but ranked lower for the technical feasibility of their introduction. The authors underlined the need for more clinical studies on a higher number of different phage lytic enzymes and phages to ensure an effective translation into novel, safe and approved antibacterial products, especially given the typically high attrition rates during clinical analysis and the currently limited number of ongoing clinical trials. Additional investments will be needed to explore and exploit the full potential.

In this minireview, we compare the advantages and hurdles of phages and phage lytic enzymes in terms of their development towards clinically approved pharmaceutical compounds. While none of them have reached clinical implementation as pharmaceuticals yet, we explain why phage lytic enzymes moved significantly faster through the development pipeline, and why unique properties of phages represent, simultaneously, their high potential, but are also the reason for a delayed development process.

## 2. Phages versus Their Lytic Enzymes as Antibacterials: An Old and Young History

Phages were discovered in the early 20th century. They have been investigated for application in phage therapy shortly after their discovery. The earliest experiments were performed by Felix D’Herelle in 1918, treating a 12-year-old boy with severe dysentery. Twenty years later, the first commercial companies, L’Oréal in Europe and Eli Lily Company in the United States, produced phage preparations for human therapy [38]. The discovery and global use of classical antibiotics as first-line antibacterials led, to a large extent, to the abandoning of phage therapy in the west. In contrast, the use of phage therapy persisted in the USSR, even when mass antibiotic production was established in the USSR by 1950. Phage research especially found ground in Georgia, where the Eliava institute (Tbilisi) was founded, which is nowadays still a global key site for phage therapy [39]. The renewed interest in the west is triggered by the global call for novel treatments of bacterial infections because of the spread of multi-drug resistant bacterial isolates and an insufficiently filled development pipeline of new antibiotics. The prospect of phage therapy remains a matter of intense debate between proponents and opponents, awaiting clinical efficacy data.

Phage lytic enzymes have been studied since the late 50 s of the 20th century, with the first biochemical characterizations of the endolysin of streptococcal phage C1 [40], the endolysins of *E. coli* phages from the T-series (T1 up to T7), selected by Max Delbrück in an effort to focus the global work of phage researchers on a standard set of phages [40,41,42,43], and *E. coli* phage λ [44]. The T4 lysozyme was a model protein for the study of protein folding [45]. In addition, the lytic activity of endolysins from phages infecting Gram-positive bacteria was thankfully used to lyse Gram-positive cells for the study of wall carbohydrate and protein components or to produce protoplasts. Only since the beginning of the 21st century, the interest in the use of phage lytic enzymes as enzyme-based antibiotics or “enzybiotics” has emerged [13,46], and the first companies started focusing on phage lytic enzymes about 10 years later.

In spite of the longer history of phage therapy, reflected by a high number of studies addressing phage therapy compared to therapy with phage lytic enzymes, the (pre)clinical evaluation of phage lytic enzymes has obviously advanced faster. While both classes of antibacterials are clearly different from existing classes of antibacterials, the standards used in the preclinical analysis of small molecule antibiotics could be more easily translated to the preclinical evaluation of phage lytic enzymes compared to phages. Furthermore, the availability of a platform for protein production, engineering and formulation into different dosage forms for an increased number of proteinaceous products (enzymes, hormones and monoclonal antibodies) registered annually to the market will facilitate entry of the phage lytic enzymes to the market [47]. Some of the unique features of phages that may leverage the therapeutic potential (discussed below) also represent hurdles that have to be tackled, resulting in a slower process featured by gradually proceeding insights to develop phages as successful antibacterials.

## 3. Bacteriophages can Replicate and Evolve

The replicative and evolvable nature of phages has been highlighted as a unique feature in terms of therapy. After infection of—and replication in—a bacterial host, a multifold of new phage particles (burst size) are produced. Yet, it has been shown that the bacterial cell number should be higher than the proliferation threshold to sustain an active multiplication. This proliferation threshold is a function of the rate at which a phage meets a bacterium, the burst size of the phage, and phage decay through inactivation or removal by the reticulo-endothelial system of spleen and liver. In other words, phage amplification is only able to compensate for phage decay above this threshold. Below this threshold, the doses of phage particles must be sufficiently high (a multiplicity of infection of 10) to ensure killing of every cell without relying on self-replication [48,49]. When active replication takes place, phages are also able to evolve by the accumulation of stochastic mutations. In combination with natural selection, this will result in co-evolved phages that respond to the development of resistance by the target bacterium. Indeed, bacteria have evolved an extensive array of mechanisms to protect themselves from phage infection, ranging from adsorption inhibition, superinfection exclusion systems, restriction-modification systems, CRISPR-Cas mediated immunity, inhibition of crucial steps of phage multiplication [50] to the recently discovered chemical molecules [51]. Bacteria are not known to have developed natural resistance against phage lytic enzymes. Infected cells are already getting resource-depleted before phage endolysins come into play. Resistance mechanisms against the earliest stages of phage infection therefore appear as most meaningful.

The potential of replication and evolution set phages apart from any other antibacterial, but consequently also apart from any existing regulatory framework that exists for the approval process of clinical trials and eventually their approval as medicinal products. The advantages of replication of phages thus also represent a significant delaying factor in the preclinical and clinical evaluation of their potential. Phage lytic enzymes do not replicate. They must be applied in sufficiently high doses as any other antibiotic to kill the bacteria before they are removed from the body.

## 4. Pharmacokinetics

The self-replication of phages results in more complex pharmacokinetics influenced by both decay and proliferation. Due to this complexity, there is still a relative lack in the understanding of pharmacokinetics of phages. Although in vivo amplification of phages has been demonstrated, the insights are dominated by mathematical modeling of in vitro infections, which does not necessarily reflect in vivo amplification [52]. The limited knowledge on phage pharmacokinetics has already contributed to the failure of phage therapy experiments, for example when dosing relied too much on the self-replicating nature of phages [48]. In contrast, phage lytic enzymes behave more as standard pharmaceutical drugs in terms of pharmacokinetics. SAL200, a *S. aureus*-specific endolysin, has a t_1/2_ between 0.04 and 0.38 h after intravenous administration in healthy volunteers. Based on the molecular weight, the authors state that renal clearance and distribution for the intravascular to the extravascular space should be minimal. Therefore, the decay of this endolysin is mainly explained by the presence of plasma proteases [21]. Other endolysins have a longer half-life (e.g., CF-301 has a half-life of 11.3 h and P128 has a half-life of 5.2 and 5.6 h for the highest doses, 30 and 60 mg/kg, respectively) [32,53]. Simpler pharmacokinetics allow easier determination of the dosing regimen of lytic enzymes in (pre)clinical analyses compared to dosing regimen of phages.

There is a general acceptance that phages and endolysins are safe, assuming they have been produced in a pharmaceutical-grade way, e.g., lacking lipopolysaccharides. No (persistent) adverse effects were observed in phase I clinical trials (Table 1 and Table 2). Massive bacterial cell lysis, induced by either phages or endolysins, may result in the release of toxin (e.g., endotoxin) that could trigger a septic shock. Yet, a recent in vitro study showed that exposure to β -lactams leads to a higher release of endotoxin compared to when exposed to two selected *E. coli* phages, even in spite of the slower mode-of-action of β-lactams. Since β-lactams are widely and safely used, this study provides comforting data regarding the endotoxin-related safety of therapeutically relevant phages [54]. When using lytic enzymes, it is required that the dosing regimen is sufficient to kill the cells, while a too high fragmentation of the cell wall must be avoided to prevent an increase in pro-inflammatory cytokines [55].

## 5. Specificity

Phages are typically characterized by a narrow spectrum. Whereas, for some species, a single phage can be identified to kill the majority of strains (e.g., phage P100 infecting *Listeria monocytogenes*), phages killing species with high clonal diversity (e.g., *Pseudomonas aeruginosa*) typically only kill a small cohort of strains of this species. The specificity is determined by the phage receptor, antiviral defense mechanisms and specific interactions with the host machinery. Due to this high specificity, a phage-*sur-mesure* approach has to be followed for each infection. A phage then has to be selected from a previously collected phage bank or a new isolation is necessary. In addition, phages can be “trained” to become more active against the infecting bacterial strain and to elicit less bacterial resistance when applied [56]. It is evident that this approach is only possible for chronic infections. This potential of a tailor-made approach is outside the range of the currently applied regulatory framework. To treat acute infections, phage cocktails composed from phages that together span the whole spectrum of strains, are proposed. Disadvantages of phage cocktails are the complex procedures and intensive research needed for the production of a suitable and stable cocktail. However, from another perspective, phage cocktails may also become unnecessary for species of which the epidemiological strains show only limited clonal diversity. Phage lytic enzymes generally have a broader specificity at the genus or species level, with a few reported endolysins being specific at the serovar level [57]. The specificity is determined by the chemical composition of the peptidoglycan, which is largely conserved at the species level, and the presence of specific epitopes that are targeted by cell wall binding domains. The broader specificity of phage lytic enzymes offers more flexibility for the treatment of both acute and chronic infections. Yet, identification of the pathogen or a prediction with a high probability still remains essential to select a custom phage lytic enzyme. Broad-spectrum antibiotics killing both Gram-positive and Gram-negative species do not require identification, but have a drastic and adverse effect on the whole microbiota, including benign flora, which is increasingly considered as non-desired [58].

## 6. Intellectual Property and the Nagoya Protocol

Patent protection is often essential to attract necessary investments. Phages are natural, ubiquitous entities that can be relatively easily isolated and have been extensively studied since their discovery. Therefore, the probability to get patent rights for a newly isolated phage is very low. In addition, there is an ongoing debate about the possibilities to seek for patent protection of naturally occurring organisms such as phages. It is therefore recommended to shift focus on downstream processes to produces phages of good quality and stability to get patent rights. [59,60]. Other approaches are directed to phage genome engineering to produce improved phages that can be patented [61,62].

In a phage-*sur-mesure* approach, a new specific phage needs to be selected for each patient. When phage cocktails are used, their composition should be continuously updated to respond to resistance development. These continuous changes are inherently in conflict with patenting law that protects a fixed product [62]. This challenging and fragile intellectual property (IP) protection makes profit-driven economic structures indecisive to invest in this avenue [59,61]. In spite of the unsuitable IP regulations, some phage preparations obtained patent protection in the US (US7507571, US7459272, US7588929, US7758856) and EU (EP1587520 B1).

An additional regulatory constraint may be the implementation of the Nagoya Protocol [63]. The Nagoya protocol is an international legislation that entered into force in October 2014 in context of the convention on biological diversity (www.cbd.int). This protocol was enacted to achieve the convention’s third objective, i.e., fair and equitable sharing of benefits arising from genetic resources procurement. In accordance with this protocol, gaining access to genetic resources of a certain country needs legislative measures that differs between one country to another. Benefits arising from using such resources shall be shared between the two parties, provider and acquirer, according to mutually approved terms. Benefits may be either in form of a monetary payment or can be non-monetary such as technology transfer. This protocol is again in conflict with the scientific realities of phage research. Both phages and their hosts are not limited to certain national borders and are ubiquitous. Strict application of the Nagoya protocol will hamper flexible and continuous updating of phage preparations with new isolates. Amendments and clarification of the Nagoya protocol will be needed [61].

Phage lytic enzymes are molecules that fit better to the current IP laws. The relative ease to get patent protection for phage lytic enzymes is reflected by the larger number of patents. Phage lytic enzymes are expected to be relatively less affected by the Nagoya protocol. A certain enzyme sequence cannot be strictly assigned to a specific phage and protein engineering is frequently used to enhance the efficacy of the native enzymes [64,65].

## 7. Regulatory Framework

Upon their reintroduction in Western medicine, phages were classified as medicinal products (EU) or drugs (USA). Nevertheless, it has been regularly confirmed by the competent authorities for medicines that the concept of phage therapy does not fit this regulation. The evolvable, self-replicating but self-limiting nature of bacteriophages being used in a way of personalized antibacterial treatment are multiple factors that differentiate phages from any antibiotic. The lack of a suitable regulatory framework has discouraged public and private funding. This is further enhanced by the narrow spectrum of phages in combination with a quick resistance development and thus the low number of eligible patients and lower potential return-on-investment [59]. Consequently, phage therapy treatments have only been performed in Western medicine under the legislation of the Helsinki declaration adopted by the 18th World Medical Association general assembly (Helsinki, Finland, June 1964) as unproven interventions in clinical practice or out of compassionate use and under the informed consent of the patient [66]. Recently, Belgium has installed a new regulatory framework that accepts phages being included as an active pharmaceutical ingredient of a magistral preparation after compliance with a number of logical provisions. A magistral preparation is defined as “any medicinal product prepared in a pharmacy in accordance with a medical prescription for an individual patient” (Article 3 of Directive 2001/83 and Article 6 quarter, § 3 of the Law of 25 March 1964). Included phages must be produced conform the provisions of an internal monograph. Each batch must be accompanied with a certificate of analysis from a Belgian approved laboratory. Phages from this batch can then be included in magistral formulas under the responsibility of a medical doctor and a pharmacist [67]. It is clear that regulatory aspects have hindered and delayed successful therapeutic innovation and evaluation of bacteriophages. There is need for an ongoing debate between phage developers and regulatory authorities on how the regulatory framework might support and offer appropriate flexibility in delineating the tests and studies to be undertaken.

Phage lytic enzymes are less specific than phages, do not replicate or evolve and thus comply better with the regulatory framework of medicinal products. Nevertheless, the new mode-of-action compared to traditional small molecule antibiotics and the proteinaceous nature (biological versus chemical) requires the setup of customized assays during the preclinical analysis. One phage lytic enzyme (Staphefekt), which is specific against *S. aureus*, has been approved under a different regulatory framework, specifically as a class 1 medical device in Europe since 2013. The lytic enzymes are formulated in a cetomacrogol-based cream and in a gel as over-the-counter treatment. The evaluation of its clinical efficacy is based on questionnaire studies, patient reviews and three reported case studies [68]. Phage lytic enzymes are also eligible for a fast track status granted by the FDA. CF-301 obtained this designation for the treatment of *S. aureus* bacteremia. This status is granted to experimental drugs to facilitate the development, and to expedite the review of drugs to treat serious or life-threatening conditions and fill an unmet medical need. The final goal is to make the drug more rapidly available for the patient, if safety and efficacy is demonstrated.

## 8. Clinical Trials

Different clinical studies of phase I, I/II and II have demonstrated the safety of phages [27,28,29,69] and phage lytic enzymes [21,32,33,68,70], which is consistent with the extensive evidence on their safety in animal models and (human) case studies (Table 1 and Table 2). To date, one randomized, double-blind, placebo-controlled phase I/II clinical trial showing efficacy of natural phages has been reported. In this study, the authors investigated a static phage-containing medicinal product against chronic *P. aeruginosa* infections causing otitis. The bacterial burden was significantly lower in phage-treated patients (*n* = 12) compared to the placebo-treated group (*n* = 12). Furthermore, no adverse effects were observed. Unfortunately, only a preliminary report has been published [69]. Clinical efficacy of phages could not be shown in other phase II trials for various technical reasons related to trial design. A study held in Bangladesh assessed the application of an oral *E. coli* phage cocktail to reduce the severity of acute bacterial diarrhea in children [28,61]. This cocktail was well tolerated but failed to improve diarrhea. The most probable reasons put forward by the authors is the lower frequency of *E. coli* as the diarrhea-causing pathogen than anticipated, and when present, *E. coli* had a lower titer than expected. In addition, there was an insufficient coverage of the infecting strains by the used bacteriophage cocktails. These elements resulted in a failed intestinal amplification and no improved clinical outcome compared to the standard care [61]. The recently terminated clinical phase II Phagoburn trial was designed to evaluate the treatment of *P. aeruginosa* and *E. coli* infected burn wounds using a phage cocktail. It was the first multi-centered trial that applied good manufacturing practices and was approved by three national health regulators (France, Belgium, and Switzerland). However, the trial was terminated prematurely due to the lack of eligible patients and insufficient efficacy of the phage cocktail. For reasons of high endotoxin content, only a diluted cocktail with a small number of phages (10–100 PFU/mL instead of the anticipated 10^9^ PFU/mL) was actually applied. Diluted stocks also tend to be more instable [29]. Recently, the clinical trial design (phase II/III) to treat urinary tract infections with the Pyophage cocktail, which is commercially available at the Eliava institute and registered in Georgia, has been published [31]. It is obvious that the particular characteristics of phages complicate the successful execution of a clinical trial. In general, an in-depth characterization of the applied phages, standard protocols on how to amplify and purify them, could improve the outcome of clinical trials [71,72,73].

In the case of phage lytic enzymes, two phase I trials (CF-301 and SAL200) and two phase I/II trails have been completed. The results of the two phase I trials show no adverse effects [21,32,33,34,35] but the data of the I/II trials are not (yet) public. Two more clinical phase II trials have been announced (Table 2) and in the near future the clinical efficacy can be evaluated. All studies are targeting *S. aureus* ranging from topical and nasal to systemic infections.

## 9. Engineered Phages and Phage Lytic Enzymes

Using a biological such as a phage or its lytic enzymes in human medicine benefits from different traits that have evolved during natural Darwinian evolution. Typically, engineering efforts aim to perform directed evolution on a lab scale to improve the characteristics of the biological. In casu, synthetic biology and protein engineering are used to increase the therapeutic potential of phages and lytic enzymes, respectively. In fact, these efforts are similar to the extensive chemical engineering of natural antibiotics, resulting nowadays in up to the 4th generation semi-synthetic antibiotics. For phage lytic enzymes, the large, existing toolbox of protein engineering methods can be used. The most commonly used method is domain swapping [11]. Shuffling of the modular composition of phage lytic enzymes comprising cell wall binding and enzymatically active domains allows for improvement of antibacterial properties such as specificity, activity, stability, and solubility. Additionally, fusion of additional modules expands or modulates their activity. Fusion of outer membrane permeabilizing peptides to phage lytic enzymes (Artilysin^®^) sensitizes Gram-negative pathogens for their bacteriolytic action [65,74]. Addition of a polycationic peptide also increased and accelerated the bactericidal effect, while reducing the required dose, for a streptococcal endolysin [64]. Compared to many other commercially available enzymes, the potential of mutagenesis has merely been exploited for phage lytic enzymes [75].

Phage engineering, especially of lytic phages, has been more cumbersome. Phages have been engineered by a wide range of methods, yet with increasing efficiency along with the emergence of synthetic biology [76,77,78]. Phage engineering has offered a way to produce new variants with expanded host range and, hence, potentially decreasing the number of phages in the cocktail needed to cover bacterial diversity [76,79,80]. It has also provided an approach to attract investment by generating patentable phage variants [80]. Phages have been also engineered to allow killing of other strains and increase efficiency against biofilm forming bacteria by insertion of bacteriocins, enzybiotics, quorum sensing inhibitors, and biofilm degrading enzymes [81,82,83,84,85]. Purification efficiency could also be improved by insertion of purification tags [86]. Longer circulation of the phage in the bloodstream has been achieved by displaying a specific protein [87,88]. Finally, phage engineering has also been used to enhance the cell-internalization efficiency, to achieve targeted delivery [89,90] and to generate non-replicative bacteriophages to control their spread and for an immune-safe product [91,92,93]. The large majority of these engineering efforts are focused to eliminate the hurdles of phage therapy. Though, since phages have a genome and are replicative, the engineering of phages may raise itself additional legislative and ethical concerns related to genetic modification. In contrast, engineered phage lytic enzymes will not elicit these concerns.

## 10. Conclusions

Spurred by the emerging and spreading multidrug resistance of human pathogens, there has been global call for novel classes of antibacterials over the last decade. Both phages and phage lytic enzymes respond to this call. As natural predators of bacteria, phages have, since their discovery, been considered as a promising candidate. Yet, significant steps in their clinical evaluation are still needed today for the approval of their use as pharmaceutical. Their unique features have complicated their preclinical and clinical analysis, requiring more intensive research, and makes them unfit with the rigid regulatory framework. This has put a significant delay on the evaluation of their potential and we still cannot confirm or refute their clinical efficacy as a pharmaceutical. Phage lytic enzymes have been considered for application in human medicine since two decades ago. They have gone through the preclinical and clinical evaluation at a much faster pace. The upcoming years will be crucial for the evaluation of phages and their lytic enzymes for their use as pharmaceutical compounds. Meanwhile, given the typically high attrition rates during clinical trials as witnessed for antibiotics and other pharmaceutical compounds, the broad community including companies and academia must keep on investing in a better understanding, design, and development of phages and phage lytic enzymes to fill the development pipeline. 

## Figures and Tables

**Table 1:** Clinical trials of bacteriophages ranked according to registry date.

Study	Registry Date	Phase	Clinical Trial Registry Number	Trial Results	Public Data
A Prospective, Randomized, Double-Blind Controlled Study of WPP-201 for the Safety and Efficacy of Treatment of Venous Leg Ulcers (WPP-201)	22 April 2008	I	NCT00663091	No adverse events were attributed to phages targeting *P. aeruginosa*, *S. aureus* and *E. coli*	Results [27]
Antibacterial Treatment against Diarrhea in Oral Rehydration Solution	10 July 2009	-	NCT00937274	Coliphages were well tolerated, but failed to improve diarrhea in children. Efficacy failure was attributed to the low frequency of *E. coli* as diarrhea etiologic agent and contribution of other pathogens such as *Streptococcus spp.* as causative agents	Results [28]
Evaluation of Phage Therapy for the Treatment of *Escherichia coli* and *Pseudomonas aeruginosa* Wound Infections in Burned Patients (PHAGOBURN)	16 April 2014	I/II	NCT02116010	Prematurely terminated due to low number of eligible patients and low efficacy of phage cocktail compared to standard of care (SOC) antibiotic. Diluted phage cocktails (10^2^ PFU/mL) were used for technical reasons. Adverse effects appeared 23% of participants compared to 53% of SOC treated group	Results [29,30]
Standard Treatment Associated with Phage Therapy Versus Placebo for Diabetic Foot Ulcers Infected by *S. aureus* (PhagoPied)	27 January 2016	I/II	NCT02664740	-	Ongoing
Ascending Dose Study of the Safety of AB-SA01 when Topically Applied to Intact Skin of Healthy Adults	2 May 2016	I	NCT02757755	-	Not available
Bacteriophages for Treating Urinary Tract Infections in Patients Undergoing Transurethral Resection of the Prostate	4 May 2017	II/III	NCT03140085	-	Trial design [31]

**Table 2:** Clinical trials of phage lytic enzymes ranked according to registry date.

Study	Registry Date	Phase	Clinical Trial Registry Number	Trial Results	Public Data
Safety and Efficacy of an Antibacterial Protein Molecule Applied Topically to the Nostrils of Volunteers and Patients	11 December 2012	I/II	NCT01746654	-	Not available
A Study to Evaluate the Safety, Pharmacokinetics and Pharmacodynamics of N-Rephasin^®^ SAL200 in Healthy Male Volunteers	16 May 2013	I	NCT01855048	No serious adverse events were reported. The AUC and Cmax increased in a greater-than-dose-proportional manner. A dosing regimen of more than 1 mg/kg was recommended as a treatment option.	Results [21]
A Placebo-Controlled, Dose-Escalating Study to Examine the Safety and Tolerability of Single Intravenous Doses of CF-301 in Healthy Subjects	8 May 2015	I	NCT02439359	A single dose of CF-301 has a low propensity to induce an inflammatory response. Long term immunological monitoring (180 days) revealed no relation between specific antibody production and hypersensitivity factors (IgE and basophils).	Results [32,33,34,35,36]
The Effect of Gladskin on Disease Severity and the Skin Microbiome, Including *Staphylococcus aureus*, in Patients with Atopic Dermatitis	21 July 2016	I/II	NCT02840955	-	Trial design [37]
Phase IIa Clinical Study of N-Rephasin^®^ SAL200	24 March 2017	IIa	NCT03089697	-	Ongoing
Safety, Efficacy and Pharmacokinetics of CF-301 vs. Placebo in Addition to Antibacterial Therapy for Treatment of *S. aureus* Bacteremia	23 May 2017	II	NCT03163446	-	Ongoing
